# Magnesium isoglycyrrhizinate ameliorates acetaminophen-induced acute liver injury with hyperglycaemia by modulating autophagy and oxidative stress in liver-resident macrophages

**DOI:** 10.3389/fphar.2026.1744099

**Published:** 2026-04-10

**Authors:** Qingfa Bu, Yuan Liang, Zibo Xu, Bozhou Pan, Jian Wang, Lei Liu, Xuhao Ni, Qi Wang

**Affiliations:** 1 Department of General Surgery, First Affiliated Hospital of Anhui Medical University, Hefei, China; 2 Department of General Surgery, Nanjing BenQ Medical Center, The Affiliated BenQ Hospital of Nanjing Medical University, Nanjing, China; 3 School of Biological Science and Medical Engineering, Southeast University, Nanjing, China

**Keywords:** acute liver injury, autophagy, hyperglycaemia, macrophage, magnesium isoglycyrrhizinate, oxidative stress

## Abstract

**Background:**

The prevalence of drug-induced acute liver injury, represented by acetaminophen (APAP), has seriously threatened human lives. Macrophages play an important role in acute liver injury; however, the treatment options remain limited. Therefore, exploring the pathogenesis of the disease and developing new treatment strategies targeted macrophages are particularly important.

**Methods:**

This study innovatively evaluates the hepatoprotective effects of magnesium isoglycyrrhizinate (MgIG) and elucidates its underlying mechanisms modulating Kupffer cell (KC) inflammation using an APAP-overdose mouse model. Hyperglycaemia was induced in C57BL/6J mice by streptozotocin treatment, and the mice were randomly divided into Saline, APAP, APAP + NAC, and APAP + MgIG groups.

**Results:**

The data demonstrated that APAP administration elicited pronounced histopathological injury and markedly upregulated serum transaminases (ALT and AST). Treatment with MgIG ameliorated these histological lesions and suppressed transaminase elevations, paralleling the effect of N-acetylcysteine (NAC). APAP induced oxidative stress in KCs, as evidenced by increased malondialdehyde (MDA) levels, decreased superoxide dismutase (SOD) activity, and a reduction in glutathione (GSH) content. Moreover, APAP exposure significantly impaired autophagy in KCs. MgIG mitigated oxidative damage and restored autophagic activity through the AMPK/AKT signalling cascade. Notably, combined treatment with MgIG and NAC produced even greater protection against APAP-induced hepatic injury.

**Conclusion:**

This study demonstrates that MgIG provides both protective and therapeutic effects in APAP-challenged mice with hyperglycaemia by modulating autophagy and oxidative stress in liver-resident macrophages.

## 1 Introduction

Drug-induced liver injury (DILI) is a common adverse drug reaction and the leading cause of acute liver failure (ALF), with severe cases resulting in substantial liver-related morbidity and mortality (Stravitz and Lee, 2019). Acetaminophen (APAP), one of the most widely used over-the-counter analgesics and antipyretics, is safe at recommended doses but can cause severe hepatotoxicity after overdose (single adult dose >10–15 g) or chronic supratherapeutic use; APAP accounts for 40%–50% of ALF cases in many developed countries. The ubiquitous presence of APAP in multisymptom and cold preparations further increases the risk of accidental overdose through polypharmacy, and certain populations (children, chronic alcohol users, malnourished individuals, and patients with preexisting liver disease) are particularly vulnerable (Lee, 2017).

Diabetes mellitus is a major and growing global health burden (Esposito et al., 2024). Hyperglycaemia, as a defining feature of diabetes, promotes systemic and tissue inflammation and alters cellular stress responses in both acute and chronic settings (Abel et al., 2024). Epidemiological and experimental data indicate that hyperglycaemia is an independent risk factor for liver injury and can exacerbate the severity of acute hepatic insults (Dey and Lakshmanan, 2013; Yang et al., 2022). In our prior work, we demonstrated that hyperglycaemia aggravates APAP-induced liver injury by amplifying macrophage-driven inflammation through oxidative stress and dysregulation of the AMPK/PI3K/AKT signalling axis (Wang et al., 2019). Despite these mechanistic insights, targeted and effective pharmacological approaches to prevent or treat APAP hepatotoxicity in the context of hyperglycaemia are lacking.

Liquorice has a long history in traditional Chinese medicine, and its extracts display a broad spectrum of biological activities, including antiviral, antimicrobial, anti-inflammatory and hepatoprotective effects (Wang et al., 2022). Magnesium isoglycyrrhizinate (MgIG) is a magnesium salt derivative enriched in 18α-glycyrrhizic acid stereoisomers derived from liquorice and has been developed as a hepatoprotective agent with anti-inflammatory, antioxidant, and immunomodulatory properties (Xie C. et al., 2015). Preclinical studies have shown that MgIG can regulate lipid metabolism to protect liver function in nonalcoholic fatty liver disease and can attenuate inflammation, oxidative stress and apoptosis in models of acute hepatotoxicity (Lai et al., 2024; Zheng et al., 2021). These findings suggest that MgIG is a promising hepatoprotective compound, but its efficacy and mechanisms in APAP-induced liver injury, particularly under hyperglycaemic conditions, remain unexplored.

Kupffer cells (KCs), liver-resident macrophages, are central mediators of sterile inflammation and play a critical role in the pathogenesis of toxin-induced hepatic injury (Ilyas et al., 2016). Upon hepatocellular damage, KCs are activated to produce chemokines and proinflammatory cytokines that recruit and polarize additional immune cells, thereby amplifying the inflammatory cascade and tissue injury (Li et al., 2017; Wen et al., 2021). Consistent with this model, hyperglycaemic mice exhibit enhanced KC activation after APAP exposure, which is characterized by increased production of IL-6 and TNF-α, reduced production of IL-10, and increased production of reactive oxygen species (ROS), ultimately contributing to aggravated hepatic injury (Wang et al., 2019).

Autophagy is a conserved lysosomal degradation pathway that removes damaged organelles and protein aggregates and thereby maintains hepatocellular homeostasis under stress. In APAP hepatotoxicity, autophagy clears damaged mitochondria and reduces ROS accumulation, thereby limiting hepatocyte death; pharmacological induction of autophagy (for example, with rapamycin) attenuates APAP-induced liver injury, whereas inhibition of autophagy exacerbates liver damage (Ni et al., 2012). Importantly, autophagy also facilitates the removal of APAP-protein adducts, a process that protects against APAP hepatotoxicity in mice (Ni et al., 2016). Metabolic stresses associated with diabetes and hyperglycaemia can impair autophagic flux, for example, through dysregulation of the AMPK‒mTOR signalling axis, thereby weakening this endogenous protective mechanism and increasing susceptibility to acute hepatic insults (Wang et al., 2020). Taken together, these data indicate that defective autophagy links metabolic stress to heightened APAP toxicity and that pharmacological restoration or enhancement of autophagy is a plausible therapeutic strategy to mitigate APAP-induced liver injury in hyperglycaemic states.

Taken together, these observations suggest that MgIG may protect against APAP-induced liver injury under hyperglycaemic conditions by dampening KC-mediated inflammation and oxidative stress and preserving or restoring autophagic function. Therefore, the principal aim of the present study was to evaluate the hepatoprotective efficacy of MgIG in a model of APAP-induced liver injury under hyperglycaemic conditions and to elucidate the underlying mechanisms, with particular emphases on inflammation, oxidative stress, and autophagy in KCs. Elucidating these mechanisms will inform the potential therapeutic application of MgIG in preventing or treating DILI in patients with dysregulated glucose metabolism.

## 2 Materials and methods

### 2.1 Animals

Six-week-old male C57BL/6J mice were procured from the Laboratory Animal Center of Nanjing Medical University. The animals were maintained in a specific-pathogen-free (SPF) environment and provided *ad libitum* access to autoclaved food and water. All procedures were approved by the IACUC of Nanjing Medical University (approval number: IACUC-2312039).

### 2.2 Animal groupings and sample allocation

C57BL/6J mice were randomly assigned to experimental groups, with n = 6 mice per group for all *in vivo* studies. Following the modelling and treatment procedures, all six mice in each group were used for terminal analyses, including serum biochemistry, histopathology, and hepatic tissue qPCR. For downstream cellular and molecular analyses requiring fresh tissue (including Kupffer cell isolation, Western blot, ELISA, immunofluorescence, and measurements of glutathione/superoxide dismutase/malondialdehyde and reactive oxygen species), cells were collected from a randomly selected subset of 3 mice per group. This randomization was applied consistently across all the experimental groups.

### 2.3 Murine model of STZ-induced hyperglycaemia and APAP-mediated acute liver injury

Streptozotocin (STZ) or vehicle control (sodium citrate buffer) was injected intraperitoneally into separate groups of 6-week-old mice at 40 mg/kg for five consecutive days. On Day 14 (9 days after the final STZ dose), tail-vein blood glucose levels were quantified. Animals with concentrations >300 mg/dL were designated the hyperglycaemic group (STZ group), whereas those receiving a vehicle injection were designated the control group (CON group). On Day 15, all the animals in the control group were given a single intraperitoneal dose of acetaminophen (APAP; 300 mg/kg in PBS) or PBS alone. Six hours post-APAP, the mice received an i.p. injection of either magnesium isoglycyrrhizinate (MgIG; 225 mg/kg) or N-acetylcysteine (NAC; 300 mg/kg). Twenty-four hours after APAP challenge, all the animals were euthanized. The mice were anaesthetized with an overdose of pentobarbital [100 mg/kg body weight, intraperitoneally (i.p.)], followed by blood collection via cardiac puncture. Blood was drawn for serum analysis, and livers were harvested for histopathological/biochemical assessments. The selected liver portions were promptly cryopreserved in liquid nitrogen, and the others were immersed in a 10% neutral pH-buffered formaldehyde solution for histopathological evaluation.

### 2.4 Liver function tests

Following whole blood centrifugation, serum was collected. Hepatic injury biomarkers (ALT and AST) were then assayed with an Olympus automated biochemical analyser (Tokyo, Japan).

### 2.5 TUNEL staining

Following deparaffinization in toluene and sequential rehydration in a graded ethanol series, apoptosis was evaluated in formalin-fixed paraffin-embedded liver sections via TUNEL staining using a fluorescence detection system (Roche, Basel) per the manufacturer’s guidelines.

### 2.6 Histological staining and immunofluorescence microscopy

After fixation in a 10% neutral-buffered formalin solution, liver samples were embedded in paraffin and sectioned at a thickness of 4 μm. The sections were deparaffinized using xylene and rehydrated through descending concentrations of ethanol. Haematoxylin and eosin (H&E) staining was applied to facilitate microscopic evaluation of hepatic morphology and inflammation. For immunohistochemical analysis, tissue sections underwent antigen retrieval, followed by incubation with a rat monoclonal anti-mouse F4/80 antibody (BD Biosciences). This was followed by treatment with a biotin-conjugated goat anti-rat IgG secondary antibody (Vector Laboratories) and detection using an avidin–biotin complex (ABC) peroxidase kit (Vector). F4/80-positive cells were then quantified in a blinded manner across ten high-power fields (HPFs) per section. To detect iNOS and CD206 in Kupffer cells (KCs), tissue sections or isolated KC preparations were fixed and blocked and then immunolabelled with rabbit anti-mouse antibodies against iNOS (Cat# 13120T, Cell Signaling Technology) and CD206 (24595T, Cell Signaling Technology), followed by treatment with a goat anti-rabbit IgG conjugated to Texas Red (Sigma‒Aldrich). Nuclei were stained with DAPI, and positive cells were quantified in ten randomly selected fields per section at ×200 magnification by blinded evaluation.

The immunofluorescence images were reanalysed using an objective, ImageJ-based approach. For each field, background subtraction was performed, followed by measurement of the integrated density for the iNOS and CD206 channels. Nuclei were counted in the corresponding DAPI channel. The per-cell fluorescence value for each field was then calculated as the integrated density divided by the DAPI count.

### 2.7 Cell isolation and culture

Kupffer cells (KCs) were isolated according to previous methods (Wang et al., 2019). Briefly, *in situ* liver perfusion was performed via the portal vein at 37 °C, first using Hanks’ Balanced Salt Solution (HBSS), followed by HBSS containing collagenase IV. Following collagenase digestion, liver tissue was excised and carefully dissociated using a 70 μm nylon strainer. Hepatocytes were collected by centrifugation at 50 *g* for 2 min, which was repeated three times. Following resuspension in HBSS, the NPC-containing supernatant was applied to a 25%/50% discontinuous Percoll gradient. Centrifugation was then performed at 1,800×g (15 min, 4 °C). The cells obtained from the Percoll gradient interface were plated in DMEM supplemented with 10% FBS, 10 mM HEPES, penicillin (100 U/mL), streptomycin (100 μg/mL), and glutamine (2 mM). Adhesion was permitted for 15 min at 37 °C. Nonadherent cells were discarded via medium exchange. KCs were cultured for 6 h post-purification, after which both the cells and their supernatants were collected for downstream analysis.

### 2.8 Western blotting analysis

Tissue and cellular proteins were extracted by homogenization with ice-cold lysis buffer containing protease and phosphatase inhibitors. The lysates were cleared by centrifugation, and 30 µg of total protein from each sample was separated using 10% SDS‒PAGE. The resolved proteins were subsequently transferred onto PVDF membranes. The membranes were blocked and then incubated at 4 °C overnight with primary antibodies from Cell Signaling Technology specific to LC3I/II (Cat# 2775S), p62 (Cat# 5114T), Beclin-1 (Cat# 3495T), ATG5 (Cat# 12994T), ATG16L1 (Cat# 8089T), ATG7 (Cat# 2631T), AMPK (Cat# 2532S), p-AMPK (Thr172) (Cat# 2531S), AKT (Cat# 9272S), p-AKT (Ser473) (Cat# 4060T), mTOR (Cat# 2972S), p-mTOR (Ser2448) (Cat# 2971S), ULK1 (Cat# 8054T), p-ULK1 (Ser555) (Cat# 5869T), and β-actin (Cat# 4967S). HRP-conjugated secondary antibodies enabled chemiluminescence detection.

Band intensities were quantified using ImageJ software (Fiji distribution). The signal for each band was measured as the integrated optical density, which was calculated from the product of the band area and its mean pixel intensity. Local background subtraction was performed by defining a rectangular region of identical size adjacent to each band, and the measured background intensity was subtracted from the band’s value. For total proteins, the background-subtracted intensity of the target band was normalized to that of the corresponding loading control (β-Actin or GAPDH) run on the same lane. For phosphorylated proteins, the intensity of the phospho-specific band was normalized to the intensity of its respective total protein band.

### 2.9 Quantitative RT‒PCR

Cells were subjected to total RNA extraction via TRIzol reagent (Invitrogen) according to the manufacturer’s instructions. Reverse transcription was performed with 500 ng of RNA using a Transcriptor Kit (Roche), followed by qPCR on a StepOnePlus™ instrument (Applied Biosystems) using SYBR Green chemistry (Roche). Following initial denaturation at 95 °C for 30 s, 40 amplification cycles were carried out (95 °C for 5 s; 60 °C for 30 s). A melt curve analysis was subsequently performed (95 °C for 15 s → 60 °C for 60 s → 95 °C for 15 s). The results of triplicate qPCRs were analysed via the 2^−ΔΔCT^ method (endogenous control: *Actb*). The primer sequences are listed in Table 1.

**TABLE 1:** Primer sequences for the amplification.

Genes	Gene forward primer (5′ → 3′)	Reverse primer (5′ → 3′)
*Tnfa*	5′-GAC​GTG​GAA​CTG​GCA​GAA​GAG-3′	5′-TTG​GTG​GTT​TGT​GAG​TGT​GAG-3′
*Il6*	5′-CCA​AGA​GGT​GAG​TGC​TTC​CC-3′	5′-CTG​TTG​TTC​AGA​CTC​TCT​CCC​T-3′
*Il10*	5′-GCT​CTT​ACT​GAC​TGG​CAT​GAG-3′	5′-CGC​AGC​TCT​AGG​AGC​ATG​TG-3′
*Ccl2*	5′-TTA​AAA​ACC​TGG​ATC​GGA​ACC​AA-3′	5′-GCA​TTA​GCT​TCA​GAT​TTA​CGG​GT-3′
*Nos2*	5′-GTT​CTC​AGC​CCA​ACA​ATA​CAA​GA-3′	5′-GTG​GAC​GGG​TCG​ATG​TCA​C-3′
*Arg1*	5′-CTC​CAA​GCC​AAA​GTC​CTT​AGA​G-3′	5′-AGG​AGC​TGT​CAT​TAG​GGA​CAT​C-3′
*Cd206*	5′-CTC​TGT​TCA​GCT​ATT​GGA​CGC-3′	5′-CGG​AAT​TTC​TGG​GAT​TCA​GCT​TC-3′
*Map1lc3a*	5′-GAC​CGC​TGT​AAG​GAG​GTG​C-3′	5′-CTT​GAC​CAA​CTC​GCT​CAT​GTT​A-3′
*Sqstm1*	5′-AGG​ATG​GGG​ACT​TGG​TTG​C-3′	5′-TCA​CAG​ATC​ACA​TTG​GGG​TGC-3′
*Becn1*	5′-ATG​GAG​GGG​TCT​AAG​GCG​TC-3′	5′-TCC​TCT​CCT​GAG​TTA​GCC​TCT-3′
*Atg5*	5′-TGT​GCT​TCG​AGA​TGT​GTG​GTT-3′	5′-GTC​AAA​TAG​CTG​ACT​CTT​GGC​AA-3′
*Atg7*	5′-GTT​CGC​CCC​CTT​TAA​TAG​TGC-3′	5′-TGA​ACT​CCA​ACG​TCA​AGC​GG-3′
*Atg16l1*	5′-CAG​AGC​AGC​TAC​TAA​GCG​ACT-3′	5′-AAA​AGG​GGA​GAT​TCG​GAC​AGA-3′
*Mtor*	5′-ACC​GGC​ACA​CAT​TTG​AAG​AAG-3′	5′-CTC​GTT​GAG​GAT​CAG​CAA​GG-3′
*Ulk1*	5′-AAG​TTC​GAG​TTC​TCT​CGC​AAG-3′	5′-CGA​TGT​TTT​CGT​GCT​TTA​GTT​CC-3′
*Actb*	5′-GGC​TGT​ATT​CCC​CTC​CAT​CG-3′	5′-CCA​GTT​GGT​AAC​AAT​GCC​ATG​T-3′

### 2.10 Measurement of hepatic superoxide dismutase (SOD) activity and glutathione (GSH) and malondialdehyde (MDA) levels

Hepatic levels of GSH and MDA, as well as SOD activity, were quantified using commercial colorimetric assay kits (GSH, Cat# A006-2-1; SOD, Cat# A001-3-2; MDA, Cat# A003-1-2) from Nanjing Jiancheng Bioengineering Institute (Nanjing, China), following the manufacturer’s guidelines.

### 2.11 Reactive oxygen species (ROS) assay

A carboxy-H_2_DCFDA fluorescent probe was used to assess intracellular ROS levels in KCs following the manufacturer’s protocol. Fluorescence imaging was performed using confocal laser scanning microscopy, and the intensity was quantified to determine the degree of ROS production.

### 2.12 ELISA

Serum and culture supernatant levels of TNF-α (Invitrogen, Cat# BMS607-3), IL-6 (Invitrogen, Cat# BMS603-2), and IL-10 (Invitrogen, Cat# BMS614) were measured using the manufacturer’s recommended protocol.

### 2.13 Statistical analysis

The data are expressed as the mean ± SD. One-way ANOVA with Bonferroni *post hoc* correction was used for multigroup comparisons, with statistical analysis conducted using STAT software and a significance threshold of *P* < 0.05 (two-tailed).

## 3 Results

### 3.1 MgIG alleviates APAP-induced acute liver injury with hyperglycaemia

**FIGURE 1:**
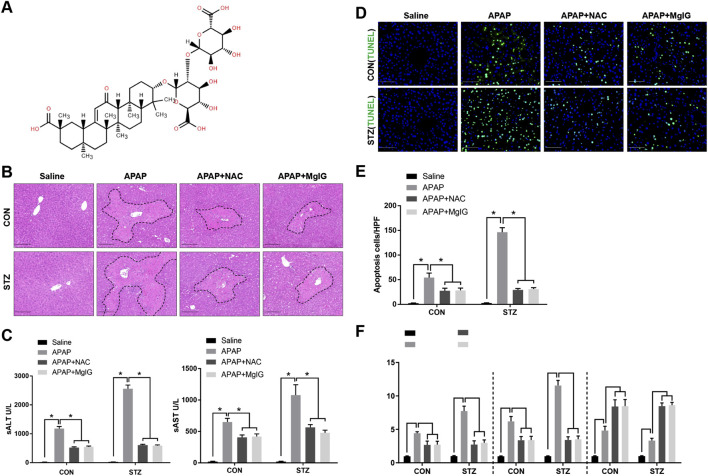
MgIG alleviates APAP-induced hepatic injury with hyperglycaemia. Hyperglycaemic mice (induced by streptozotocin, STZ) and normoglycaemic controls (CON) were established following the protocols detailed in the “Materials and Methods” section. Hepatic injury was induced by APAP administration, whereas control animals underwent a sham intervention. MgIG or NAC was used to treat the above experimental groups. **(A)** Chemical structure of magnesium isoglycyrrhizinate. **(B)** Histopathological assessment of APAP + STZ-induced liver injury with or without MgIG or NAC treatment. The necrotic areas, characterized by loss of cellular architecture and inflammatory infiltration, are demarcated by dashed lines. Representative images from six mice per group are shown. Scale bars, 100 μm. **(C)** Plasma levels of ALT and AST were determined 24 h after APAP + STZ administration with or without MgIG or NAC treatment (n = 6/group). **(D)** Apoptotic hepatocytes were identified by TUNEL staining of liver tissue sections, with nuclei counterstained using DAPI. Representative images from six mice per group are provided. Scale bars, 50 μm. **(E)** The number of TUNEL-positive cells was quantified across groups (n = 6/group). **(F)** Hepatic mRNA expression of proinflammatory genes was analysed using quantitative real-time PCR (n = 6/group) (mean ± SD, ^*^ *P* < 0.05).

Similar to NAC treatment, MgIG treatment significantly attenuated APAP-induced liver injury in both normoglycaemic and STZ-induced hyperglycaemic mice (Figure 1A). H&E staining revealed reduced centrilobular necrosis in MgIG- or NAC-treated livers (dashed circles), which was confirmed by a decreased percentage of necrotic area compared with those in the APAP and STZ + APAP groups (Figure 1B). This improvement was supported by significantly reduced serum ALT and AST levels (Figure 1C), decreased hepatoapoptosis (Figures 1D,E) and decreased proinflammatory gene expression (Figure 1F). Collectively, these findings indicate that MgIG or NAC treatment attenuated APAP-induced hepatic injury comorbid with hyperglycaemia.

### 3.2 MgIG inhibits the progression of inflammation and mediates KC anti-inflammatory polarization in APAP-induced hepatic injury with hyperglycaemia

**FIGURE 2:**
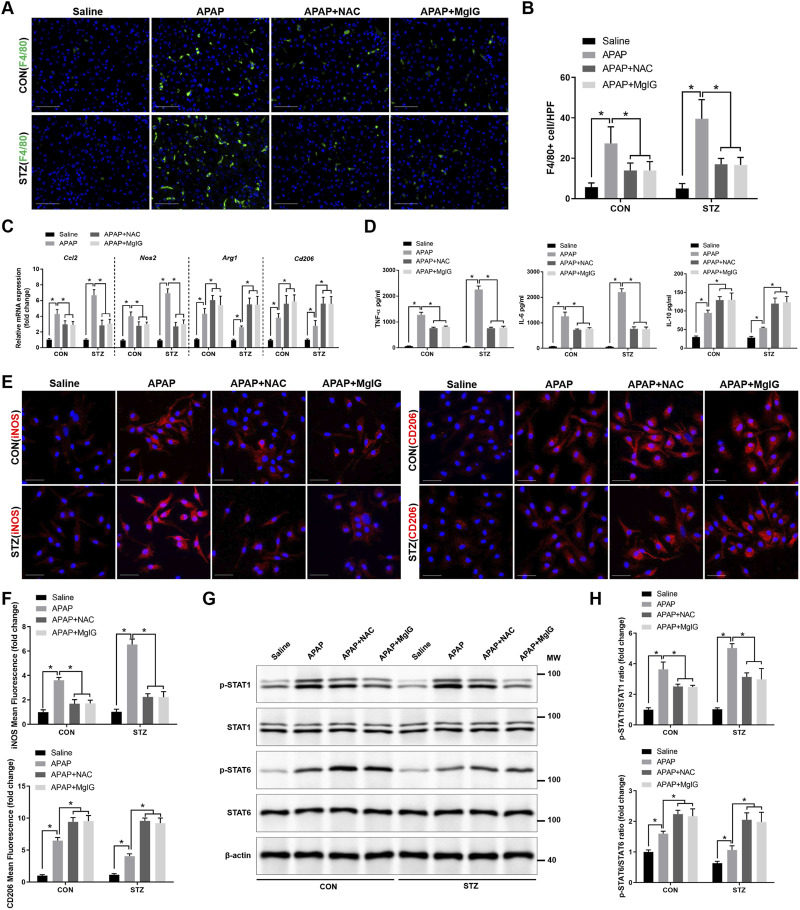
MgIG inhibits inflammation progression and promotes the anti-inflammatory polarization of KCs in APAP-induced hepatic injury with hyperglycaemia. **(A,B)** F4/80^+^ macrophages in the liver were visualized via immunofluorescence microscopy at ×200 magnification. Images representative of six mice per group are presented. Quantitative analysis of F4/80^+^ cell counts was performed per high-power field (n = 6/group). Scale bars, 50 μm. **(C)** mRNA levels of markers associated with proinflammatory (*Ccl2*, *Nos2*) and anti-inflammatory (*Arg1*, *Cd206*) phenotypes were assessed using real-time quantitative PCR (n = 3/group). **(D)** KCs harvested from the indicated groups were cultured for 6 h, after which the concentrations of TNF-α, IL-6, and IL-10 in the culture media were determined using ELISA (n = 3/group). **(E)** iNOS and CD206 expression in KCs was assessed by immunofluorescence imaging, with DAPI counterstaining of the nuclei. The images shown are representative of three independent replicates. Scale bars, 20 μm. **(F)** The percentage of cells expressing iNOS or CD206 was calculated for each group (n = 3/group). **(G)** Immunoblot analysis was conducted to determine the protein levels of phosphorylated and total STAT1, STAT6, and β-actin. The blots displayed are representative of three separate experiments. **(H)** Results from the quantitative analysis of STAT1 and STAT6 phosphorylation (p-STAT1/STAT1 and p-STAT6/STAT6 ratios) from the blots shown in **(G)** (mean ± SD, ^*^ *P* < 0.05).

Given that acute liver injury is closely associated with an inflammatory response, the total intrahepatic macrophage counts in APAP-treated livers from hyperglycaemic mice, with or without MgIG or NAC treatment, were measured across different groups using F4/80 immunofluorescence. The results demonstrated that MgIG or NAC treatment significantly decreased F4/80^+^ macrophage infiltration in APAP-treated hyperglycaemic mice (Figures 2A,B). Macrophages are generally categorized into proinflammatory and anti-inflammatory types based on their functions (Brenner et al., 2013). Therefore, we investigated how hyperglycaemia influences the polarization of KCs towards proinflammatory and anti-inflammatory states. Compared with KCs in the untreated model groups (APAP, STZ + APAP), those from MgIG- or NAC-treated hyperglycaemic mice showed reduced expression of proinflammatory genes (*Ccl2* and *Nos2*) and increased expression of anti-inflammatory markers (*Arg1* and *Cd206*) (Figure 2C). KC supernatants from the treated groups showed suppressed proinflammatory cytokine secretion (TNF-α and IL-6) but increased anti-inflammatory IL-10 production compared with those from the control group, as quantified by ELISA (Figure 2D). Compared with untreated APAP and STZ + APAP controls, MgIG or NAC treatment significantly reduced iNOS^+^ proinflammatory Kupffer cells while increasing CD206^+^ anti-inflammatory KCs in hyperglycaemic mice post-APAP challenge (Figures 2E,F). Furthermore, Western blotting (Figures 2G,H) revealed that MgIG or NAC treatment attenuated STAT1 phosphorylation and increased STAT6 phosphorylation in KCs isolated from APAP-treated hyperglycaemic mice. These results indicated that MgIG or NAC treatment inhibits inflammatory progression by suppressing proinflammatory polarization and promoting the anti-inflammatory polarization of KCs in APAP-induced hepatic injury with hyperglycaemia.

### 3.3 MgIG alleviates APAP-induced hepatic injury with hyperglycaemia by suppressing AMPK/AKT-mediated oxidative stress in KCs

**FIGURE 3:**
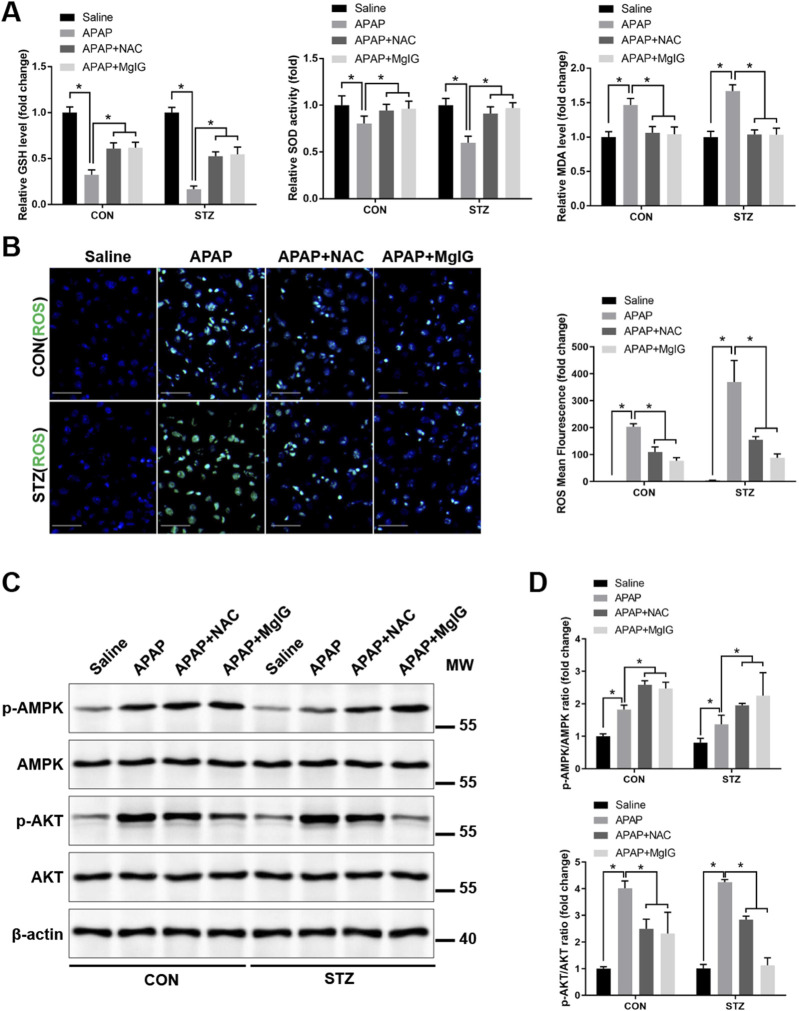
MgIG alleviates APAP-induced hepatic injury with hyperglycaemia by suppressing AMPK/AKT-mediated oxidative stress in KCs. **(A)** Hepatic oxidative stress was evaluated via quantification of SOD activity and GSH and MDA contents using commercially available detection kits. **(B)** Intracellular ROS levels in KCs were visualized with the fluorescent probe carboxy-H_2_DFFDA. The images reflect the results from three separate experimental replicates. The numbers of fluorescence-positive cells in 10 randomly selected high-power fields per section were quantified. Scale bars, 20 μm. **(C)** The expression of p-AMPK, AMPK, p-AKT, AKT, and β-actin in KCs from each treatment group was determined by Western blot analysis. The blots shown are indicative of findings from three independent assays. **(D)** Quantitative analysis of AMPK and AKT phosphorylation in Kupffer cells (mean ± SD, ^*^ *P* < 0.05).

Our previous study demonstrated that hyperglycaemia drives ROS generation in KCs during APAP exposure by suppressing AMPK signalling and concurrently activating AKT signalling (Wang et al., 2019). To determine how MgIG alleviates hyperglycaemia-associated APAP hepatotoxicity, we evaluated oxidative stress in KCs. The results indicated that MgIG treatment markedly increased the level of the antioxidant GSH and the activity of the antioxidant enzyme SOD but suppressed the level of the lipid peroxidation product MDA (Figure 3A). The effects of NAC were similar to those of MgIG in the APAP and STZ + APAP groups. Based on these findings, we measured intracellular ROS levels in KCs obtained from hyperglycaemic mice treated with APAP ± MgIG or NAC. The results revealed that hyperglycaemia significantly increased ROS production in KCs (Figure 3B), whereas treatment with MgIG or the ROS inhibitor NAC significantly reduced ROS levels in both the APAP and STZ + APAP groups (Figure 3B). Western blotting revealed that MgIG or NAC treatment significantly increased AMPK phosphorylation and decreased AKT phosphorylation in KCs isolated from APAP-treated hyperglycaemic mice (Figures 3C,D). These findings demonstrated that MgIG alleviates APAP-induced hepatic injury with hyperglycaemia by suppressing AMPK/AKT-mediated oxidative stress in KCs.

### 3.4 MgIG ameliorates APAP-induced hepatic injury with hyperglycaemia by restoring autophagic activity in KCs

**FIGURE 4:**
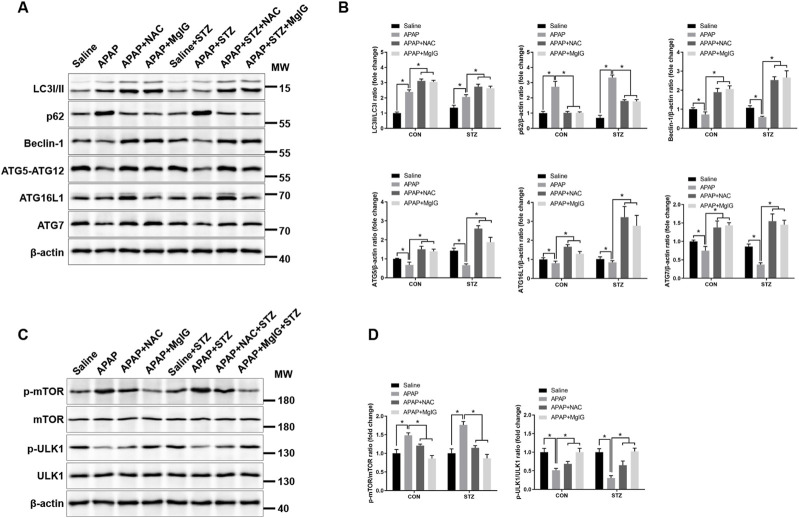
MgIG ameliorates APAP-induced hepatic injury with hyperglycaemia by suppressing autophagy in KCs. **(A)** Western blotting was used to assess LC3 I/II, p62, Beclin-1, ATG5-ATG12, ATG16L1, and ATG7 protein levels in KCs obtained from the respective groups. **(B)** Quantification of autophagy-related proteins from **(A)**. **(C)** Phosphorylated mTOR and ULK1 protein levels in KC samples from different treatment groups were evaluated by immunoblotting. **(D)** The relative expression ratios of p-mTOR to total mTOR and p-ULK1 to total ULK1 were quantified in KCs (mean ± SD, ^*^ *P* < 0.05).

Our previous study demonstrated that hyperglycaemia aggravates hepatic inflammation in acute liver injury via impaired mTOR-regulated autophagy in KCs (Wang et al., 2020). To further determine whether MgIG exerts its protective effect by modulating KC autophagy, we evaluated the expression of autophagy-related markers. The data in Figures 4A,B demonstrate that MgIG upregulated the protein expression levels of autophagy mediators (LC3, Beclin-1, ATG5-ATG12, ATG16L1, and ATG7) but downregulated the expression of p62 in KCs from APAP-exposed hyperglycaemic mice. To determine the impact of MgIG on autophagy regulation, we assessed mTOR and ULK1 expression at the transcriptional and translational levels. MgIG suppressed mTOR phosphorylation (p-mTOR) while activating ULK1 (p-ULK1) in KCs from hyperglycaemic APAP-exposed mice, indicating enhanced autophagic initiation (Figures 4C,D). These data indicate that MgIG alleviates APAP-induced hepatic injury under hyperglycaemic conditions by reactivating Kupffer cell autophagy.

### 3.5 Autophagy or AMPK inhibition reverses the ability of MgIG to prevent APAP-induced acute liver injury with hyperglycaemia

**FIGURE 5:**
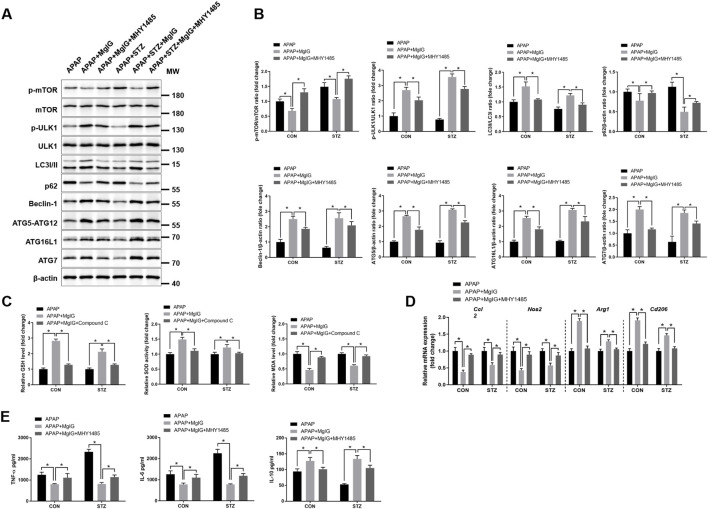
Autophagy or AMPK inhibition abolished the hepatoprotective effects of MgIG against APAP-induced hepatic injury with hyperglycaemia. Control and STZ-induced hyperglycaemic mice were pretreated *in vivo* with the mTOR activator MHY1485 or the AMPK inhibitor Compound C prior to APAP administration (detailed in the Materials and Methods). **(A)** The protein expression of LC3 I/II, p62, Beclin-1, ATG5-ATG12, ATG16L1, and ATG7 in KCs isolated from each group was measured by Western blotting. **(B)** Quantification of autophagy-related proteins from **(A)**. **(C)** Oxidative stress in the liver was evaluated by measuring GSH and MDA levels and SOD activity using commercial assay kits. **(D)** The mRNA expression levels of proinflammatory markers (*Ccl2* and *Nos2*) and anti-inflammatory markers (*Arg1* and *Cd206*) in KCs were quantified via qRT‒PCR (n = 3/group). **(E)** KCs from the respective groups were incubated for 6 h, after which the concentrations of cytokines, including TNF-α, IL-6, and IL-10, in the culture supernatant were quantified using ELISA (n = 3/group) (mean ± SD, ^*^ *P* < 0.05).

To determine whether the hepatoprotection effect of MgIG requires autophagy activation and AMPK/AKT redox regulation, we pharmacologically inhibited these pathways using MHY1485 (mTOR activator/autophagy blocker) and Compound C (AMPK inhibitor). MHY1485 abolished MgIG-induced autophagy activation in KCs from APAP/hyperglycaemic mice; suppressed the expression of LC3, Beclin-1, Atg5, Atg16L1, and Atg7; and increased p62 accumulation (Figures 5A,B). Moreover, Compound C abrogated the antioxidant effects of MgIG in these KCs, as it suppressed GSH levels and SOD activity but increased MDA levels (Figure 5C). Additionally, MHY1485 altered KC polarization by increasing the mRNA expression of *Ccl2* and *Nos2* and decreasing the expression of *Arg1* and *Cd206* (Figure 5D). It also promoted the secretion of TNF-α and IL-6 while reducing the secretion of IL-10 in culture supernatants (Figure 5E). These results demonstrated that the inhibition of autophagy or AMPK signalling reversed the anti-inflammatory effects of MgIG and negated its ability to prevent APAP-induced acute liver injury with hyperglycaemia. MHY1485 (10 mg/kg), an mTOR activator/autophagy blocker, was administered 1 h before APAP injection. Compound C (10 mg/kg), an AMPK inhibitor, was administered 0.5 h before APAP injection.

### 3.6 MgIG and NAC in combination exert an enhanced protective effect against APAP-induced hepatic injury with hyperglycaemia

**FIGURE 6:**
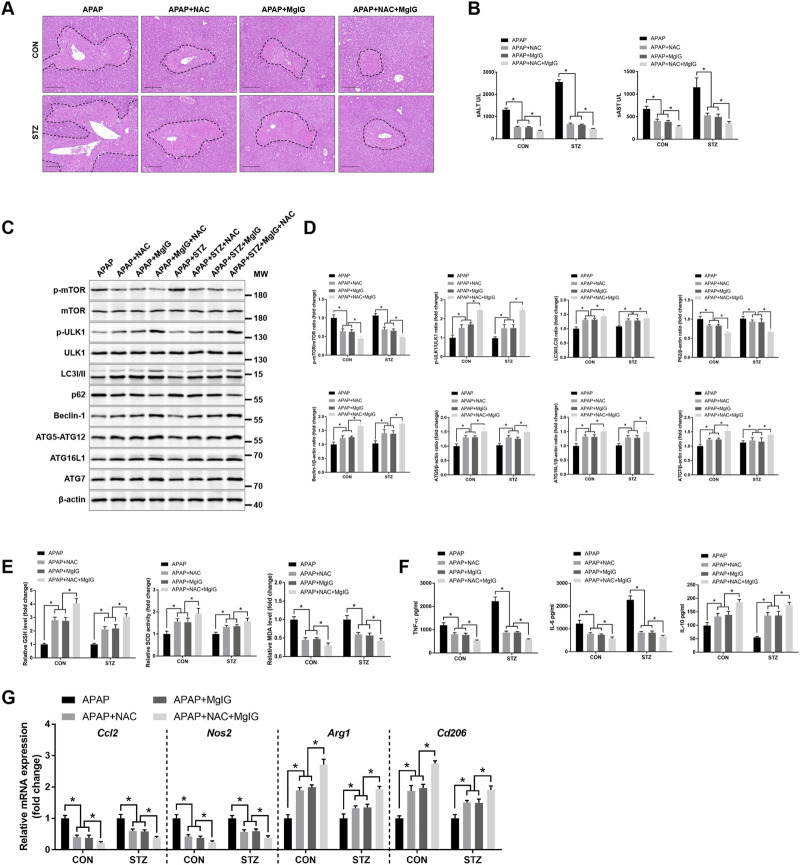
The combination of MgIG and NAC further ameliorated APAP-induced hepatic injury with hyperglycaemia. STZ-induced hyperglycaemic and normoglycaemic controls received pretreatment with MgIG, NAC, or a combination of MgIG + NAC, followed by APAP administration (detailed in the Materials and Methods). **(A)** Representative H&E-stained liver sections from each treatment group; dashed circles indicate centrilobular/coagulative necrotic zones. Images are representative of six mice per group. Scale bars, 100 μm. **(B)** Serum concentrations of ALT and AST were determined (n = 6/group). **(C)** The protein expression of p-mTOR, p-ULK1, LC3 I/II, p62, Beclin-1, ATG5-ATG12, ATG16L1, and ATG7 in KCs isolated from each group was measured by Western blotting. **(D)** Quantification of autophagy-related proteins from **(C)**. **(E)** Oxidative stress in the liver was evaluated by measuring GSH and MDA levels, and SOD activity using commercial assay kits. **(F)** KCs from each group were cultured for 6 h, and the concentrations of cytokines, including TNF-α, IL-6, and IL-10, in the culture supernatant were quantified using ELISA (n = 3/group). **(G)** The mRNA expression levels of proinflammatory markers (*Ccl2* and *Nos2*) and anti-inflammatory markers (*Arg1* and *Cd206*) in KCs were quantified via qRT‒PCR (n = 3/group) (mean ± SD, ^*^ *P* < 0.05).

Compared with NAC monotherapy, the combination regimen resulted in significantly less histopathological liver injury and lower serum ALT and AST levels (Figures 6A,B). H&E-stained sections revealed that compared with monotherapy, the combination treatment resulted in centrilobular necrotic foci of reduced size, indicating superior therapeutic efficacy. Moreover, combination therapy significantly increased autophagic activity in KCs isolated from treated livers, as evidenced by elevated expression levels of autophagy-related proteins (LC3, Beclin-1, ATG5-ATG12, ATG16L1, and ATG7) and a concomitant reduction in p62 protein levels (Figures 6C,D). Additionally, the combination therapy restored antioxidant defences, as evidenced by elevated GSH levels and SOD activity and reduced MDA levels (Figure 6E). We subsequently examined Kupffer cell polarization. The results demonstrated that the combination therapy significantly decreased TNF-α and IL-6 secretion while increasing IL-10 release (Figure 6F). qPCR analysis of hepatic KCs corroborated these findings, revealing downregulation of *Ccl2* and *Nos2* mRNA expression and upregulation of *Arg1* and *Cd206* expression in APAP-exposed hyperglycaemic mice compared with those in mice treated with NAC monotherapy (Figure 6G). Taken together, these findings indicate that MgIG and NAC coadministration provides enhanced hepatoprotection against APAP-induced hepatic injury in hyperglycaemic mice, which is associated with a more robust promotion of autophagy, increased antioxidant capacity, and Kupffer cell repolarization towards an anti-inflammatory phenotype.

## 4 Discussion

Diabetes mellitus, characterized by chronic hyperglycaemia, is a global health burden that substantially compromises patient quality of life (Lyssenko and Vaag, 2023). If left untreated, diabetes can lead to various macrovascular and microvascular complications, such as arterial plaque buildup (Poznyak et al., 2020), kidney damage (Gross et al., 2005), retinal degeneration (Fong et al., 2004), and nerve dysfunction (Bronson and ReiterGardner, 2003). We examined the effect of hyperglycaemia on APAP-induced hepatic injury, with a particular focus on the proinflammatory activation of KCs. We provide the first evidence that MgIG confers hepatoprotection against APAP injury in hyperglycaemia through dual modulation of hepatic macrophages: inducing autophagy and counteracting oxidative stress in KCs.

APAP-induced hepatic injury serves as a cornerstone model in drug toxicology; however, the key mechanisms involved in the manifestation of this type of injury remain incompletely understood. In the liver, APAP is metabolized by cytochrome P450 enzymes, producing the metabolite NAPQI (Li et al., 1994). The depletion of GSH enables NAPQI to bind to cellular proteins, thereby initiating oxidative stress and mitochondrial dysfunction (James et al., 2003). Critically, the activation of the mitochondrial permeability transition, excessive ROS generation, and DNA damage collectively trigger necrotic hepatocyte death and the propagation of inflammatory cascades (Robichaux et al., 2023). DAMPs liberated from necrotic hepatocytes engage KCs and neutrophils, triggering the release of proinflammatory cytokines (e.g., TNF-α and IL-1β) (Kaltenmeier et al., 2022). Genetic polymorphisms (such as CYP2E1 variants) (Sridharan et al., 2023), comorbidities (such as alcoholic liver disease) (Ge et al., 2023), and nutritional status (including GSH reserves) influence toxicity thresholds (Sriphoosanaphan et al., 2023); however, predictive biomarkers for personalized risk assessment are lacking.

Exhibiting substantial functional plasticity, KCs adopt a spectrum of phenotypes, from classically activated (M1) to alternatively activated (M2), that dictate hepatic inflammatory outcomes (Koyama and Brenner, 2017). M1 macrophage activation is characterized by increased expression of STAT1 and IRF-5, key regulators of proinflammatory gene transcription (Merecz-Sadowska et al., 2020), accompanied by increased secretion of TNF-α, IL-6, and IL-12, along with increased expression of iNOS (Shapouri-Moghaddam et al., 2018). In contrast, M2 polarization is defined by the expression of STAT6, IRF-4, and PPARγ and the production of IL-10 (Merecz-Sadowska et al., 2020). Subsequent studies have indicated that the inactivation of KCs with gadolinium chloride and dextran sulphate can attenuate APAP-induced hepatic injury in mice, which occurs concomitantly with the loss of nitrotyrosine staining. Gadolinium chloride and its analogues inhibit KC activity, which is primarily achieved by impairing their ability to produce ROS (Michael et al., 1999). These studies suggest that KCs represent a major contributor to ROS generation after APAP overdose.

N-acetylcysteine, the only treatment authorized by the FDA for APAP overdose, is effective only if it is administered within 8–10 h after an overdose occurs (Qiao et al., 2012). Late-stage patients often require liver transplantation due to irreversible damage. MgIG, which is a stereoisomer of glycyrrhizic acid derived from liquorice root, has demonstrated significant hepatoprotective effects via multiple mechanisms. For example, MgIG inhibits NF-κB and MAPK signalling, resulting in decreased production of proinflammatory mediators and reduced hepatic inflammation (Wang et al., 2018). MgIG also increases the expression of key cellular redox defence components, including SOD and GSH-Px, via activation of the NRF2/ARE pathway, thus mitigating oxidative stress in hepatocytes. Additionally, MgIG regulates the expression of apoptosis-related proteins (Bcl-2 and Bax) and suppresses caspase-3 activation, thus preventing hepatocyte apoptosis (Tan et al., 2018). Recently, MgIG has been shown to be effective at alleviating acute liver injury (Fan et al., 2021). However, no studies have explored how MgIG influences APAP-induced hepatic injury or clarified the associated molecular pathways.

In multicellular organisms, ROS are indispensable for ontogenesis, cellular expansion, and differentiation, with homeostatic concentrations permitting essential proliferative/metabolic activities. Notably, ROS also constitute evolutionarily conserved signalling mediators that coordinate diverse metabolic and regulatory pathways (Song et al., 2020). Recent evidence has established oxidative stress as a key driver of diabetic complications (Giacco and Brownlee, 2010). Evidence confirms that hyperglycaemia drives ROS production in macrophages (Veluthakal et al., 2024). In diabetes, monocytes are recruited by MCP-1 secreted from the endothelium (Whetzel et al., 2006). Recruited monocytes differentiate into macrophages that drive ROS overproduction, exacerbating inflammation and tissue damage (Brenner et al., 2013). Additional research has demonstrated that hyperglycaemia increases ROS production in macrophages via mitochondrial dysfunction and the aberrant activation of cytoplasmic NADPH oxidases (NOXs) (Lu et al., 2020). Unlike the tightly regulated ROS bursts that are characteristic of antimicrobial responses, diabetic macrophages exhibit erratic, dysregulated ROS production from metabolic sources. This aberrant signalling promotes an M1-like phenotype, worsening diabetic complications (Zhao et al., 2020). It has been hypothesized that APAP metabolism triggers oxidative stress (Xie Y. et al., 2015). Accumulating evidence has confirmed that the generation of mitochondrial ROS is a critical driver of APAP-induced hepatotoxicity (Deng et al., 2024).

As a key adaptive mechanism, autophagy enables cells to degrade and recycle misfolded proteins and defective organelles via lysosome-mediated pathways in response to various stress signals (Boya and ReggioriCodogno, 2013; Kim and Lee, 2014). Despite the limited understanding of macrophage autophagy in the context of liver diseases, emerging data demonstrate its protective role against acute hepatic injury. In aged mice, the knockout of ATG4B, an autophagy-related protein, significantly worsened liver ischaemia‒reperfusion injury (Wang et al., 2011). Autophagy within macrophages has hepatoprotective effects against acute toxin-induced injury, reducing mortality through the inhibition of IL-1β production (Ilyas et al., 2016). Conversely, a deficiency in hepatic autophagy can impair FXR function and lead to cholestatic injury (Khambu et al., 2019). Our earlier findings revealed that the activation of autophagy in KCs inhibits proinflammatory polarization, promotes anti-inflammatory polarization, and attenuates TAA-induced hepatic injury (Wang et al., 2020). This study revealed that MgIG promoted KC autophagy and suppressed KC proinflammatory polarization.

Notably, as highlighted by recent guidelines (Klionsky et al., 2021), the assessment of autophagic activity is most accurately achieved through the measurement of autophagic flux, the dynamic process from autophagosome formation to lysosomal degradation. In the present study, we inferred altered autophagic activity based on well-established markers, including an increased LC3-II/LC3-I ratio and decreased p62 protein levels in KCs. However, we acknowledge that without direct flux analysis (e.g., using lysosomal inhibitors or tandem fluorescent LC3 reporters), our conclusions regarding the dynamics of autophagy remain indirect. This represents a limitation of our current work. Future studies employing such definitive flux assays, as elegantly demonstrated in other metabolic contexts (Garcia-Macia et al., 2021), will be crucial to precisely delineate the role and regulation of autophagy in MgIG-mediated hepatoprotection within the complex milieu of hyperglycaemia and drug-induced injury.

NAC is widely recognized as a precursor for glutathione synthesis and a direct ROS scavenger. Its primary effect on KCs is likely the restoration of redox balance, which indirectly dampens ROS-triggered proinflammatory signalling (Aldini et al., 2018). Its activity is thus centred on antioxidant reconstitution. MgIG, our compound of interest, has been shown to exert hepatoprotective effects through mechanisms that may include the upregulation of IL-22 and the increase in autophagy, leading to more direct suppression of the TLR4–NF-κB inflammatory pathway in liver injury models (Zhou et al., 2022). Its action appears to be more targeted towards modulating specific inflammatory and autophagic pathways. Although their final phenotypic outputs (reduced oxidative stress and inflammation) overlap, their primary molecular targets and immediate modes of action differ. This mechanistic divergence provides a rationale for their potentially complementary or additive effects when used in combination, as they may intercept the injury cascade at different nodes.

## 5 Conclusion

Overall, our study demonstrated that MgIG alleviated liver pathology and reduced the circulating levels of ALT and AST in APAP-exposed mice, with these effects being similar to those of NAC. MgIG also attenuated oxidative stress in KCs obtained from the livers of APAP-exposed mice, as evidenced by reduced MDA concentrations, increased SOD activity and increased GSH levels. Furthermore, MgIG upregulated the expression of key autophagy-related genes at both the transcriptional and translational levels. In parallel, p-mTOR protein levels were decreased, whereas p-ULK1 protein levels were increased in KCs. MgIG also attenuated oxidative injury by activating the AMPK/AKT pathway. Following MgIG administration, Beclin-1, LC3-II, Atg5, Atg16L1, and Atg7 expression was normalized to baseline levels. Finally, the combination of MgIG and NAC provided even greater protection against hyperglycaemia-exacerbated APAP hepatotoxicity. In conclusion, MgIG has therapeutic effects on APAP-induced liver injury through the modulation of KC autophagy and oxidative stress.

## Data Availability

The raw data supporting the conclusions of this article will be made available by the authors, without undue reservation.
